# Hospital Advertising

**Published:** 1914-06-20

**Authors:** Charles Cutting

**Affiliations:** 4 St. Paul's Churchyard, E.C.


*To the Editor of* The Hospital.

### Hospital Advertising.

*To the Editor of* The Hospital.

Sir,—I look upon The Hospital as an old friend and, naturally, I like most to see it at its best. May I jot down two brief quotations and one or two equally brief comments on some remarks which I find on page 284 of the issue dated June 13, 1914?

"Any of our readers who happened to study our Hospital Sunday Number last week will have observed that every contributor from the Chief Rabbi to the leader-writer was not beating a hollow drum when insisting on the religious inspiration which, as a fact, does come to those people who are engaged in personal service and institutional work."

The other quotation is:

"This means to encourage visiting, to found a variety of subsidiary organisations, to make his hospital the centre of a circle of instructed enthusiasm." Why a "hollow" drum? Could any adjective have been more unfortunately chosen? Is there any sort of drum in any land that is not hollow? Why "instructed" enthusiasm? Any advertising baby knows that the more enthusiasm is instructed the weaker it becomes. Enthusiasm is (to me) wonderful, but "instructed enthusiasm" must be very funny. Forgive me for saying that I think all the notes in your issue of the 13th on the art of advertising are laughably "mixed"; where there is no "mixing" there is staleness.—I am, your obedient servant,

Charles Cutting.

4 St. Paul's Churchyard, E.C., June 15, 1914.

[It is somewhat ludicrous that our correspondent, who "looks upon The Hospital as an old friend," should be moved to betray the fact by an objection to two adjectives which he thinks he could have chosen better himself. In common with many Englishmen he evidently does not know that the mixed metaphor is a favourite characteristic of the masters of English style. Only those who have never got beyond the *pons asinorum* of the split infinitive ever apologise for its use. To do so would betray a lack of humour. Ophelia's phrase I "sucked the honey of his music vows" is a Shakespearean example. As to his objection to "instructed enthusiasm," who but "an advertising baby" would suppose that the more "instructed" enthusiasm grows the weaker it becomes? Enthusiasm begins as vapour; it has to be converted into steam if it is to coin guineas, and not collapse at the first difficulty. Florence Nightingale was an example of instructed enthusiasm, and it is to winnow out those without this quality that we possess preliminary training schools to-day. The same process as that provided by these schools for probationers is necessary in other departments of hospital life.—Ed., The Hospital.

